# ﻿Studies of Vietnamese Pteridophyte Flora 3

**DOI:** 10.3897/phytokeys.255.141395

**Published:** 2025-04-17

**Authors:** Cheng-Wei Chen, Van Truong Do, Quang Cuong Truong, Viet Dai Dang, Hong Truong Luu, Yi-Shan Chao, Yao-Moan Huang, Kuo-Fang Chung

**Affiliations:** 1 Biodiversity Program, Taiwan International Graduate Program, Academia Sinica and National Taiwan Normal University, Taipei 115, Taiwan; 2 Biodiversity Research Center, Academia Sinica, Taipei 115, Taiwan; 3 Department of Life Science, National Taiwan Normal University, Taipei 106, Taiwan; 4 Vietnam National Museum of Nature, Vietnam Academy of Science and Technology, Hanoi, Vietnam; 5 International Center for Tropical Highlands Ecosystems Research of Bidoup - Nui Ba National Park, Lam Dong, Vietnam; 6 Institute of Advanced Technology, Vietnam Academy of Science and Technology, Ho Chi Minh City, Vietnam; 7 Taiwan Forestry Research Institute, Taipei 100, Taiwan

**Keywords:** Chromosome number, cytology, Indochina, new records, phylogeny

## Abstract

This is the third paper in a series dedicated to updating the knowledge of the Vietnamese pteridophyte flora. Based on recent collections, we first reported three new national records of ferns: *Haplopterisyakushimensis*, *Lindsaeakohkongensis*, and *Pterispseudowulaiensis*. Secondly, we conducted phylogenetic analyses to investigate the placements of *Lindsaeakohkongensis* and *Leptochiluspoilanei*, each based on three plastid DNA markers. Our results revealed that *Lindsaeakohkongensis* is sister to *L.ensifolia*, while *Leptochiluspoilanei* is embedded within *L.cantoniensis*. We discussed these results in the context of systematics. Lastly, we reported chromosome numbers for 20 fern species in Vietnam. For seven of these species, including *Gymnosphaerasalletii*, *Lepisorusspicatus*, *Leptochiluspoilanei*, *Pteridryscostularis*, *Pterislatipinna*, *Pyrrosiaeberhardtii*, and *Tectariasetulosa*, these counts were recorded for the first time. Additionally, three new cytotypes were identified for *Diplaziumdoederleinii*, *Pterisesquirolii*, and *Tectariaharlandii*. This study underscores the need for more diverse data, including DNA sequences, chromosome numbers, and reproductive modes, to be collected and integrated into systematic studies and taxonomic treatments to enhance our understanding of Vietnam’s pteridophyte flora.

## ﻿Introduction

**Figure 1.**
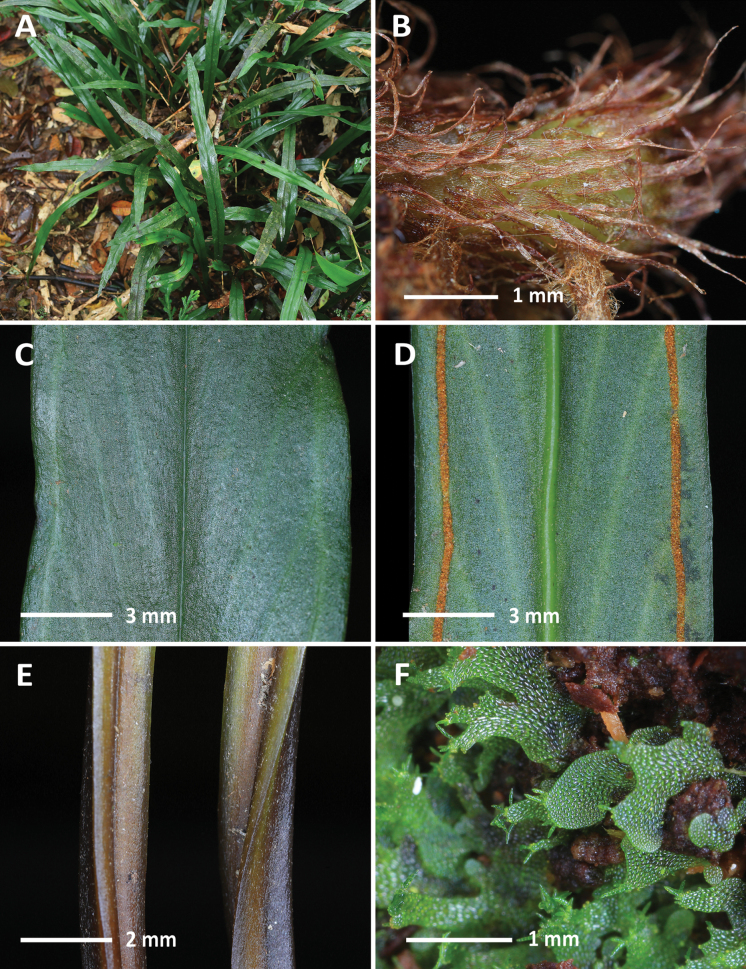
*Haplopterisyakushimensis* C.W.Chen & Ebihara (*Chen Wade6952*) **A** habit **B** rhizome scales **C** adaxial lamina **D** abaxial lamina **E** stipes **F** gametophytes. Photographed by C.-W. Chen.

**Figure 2.**
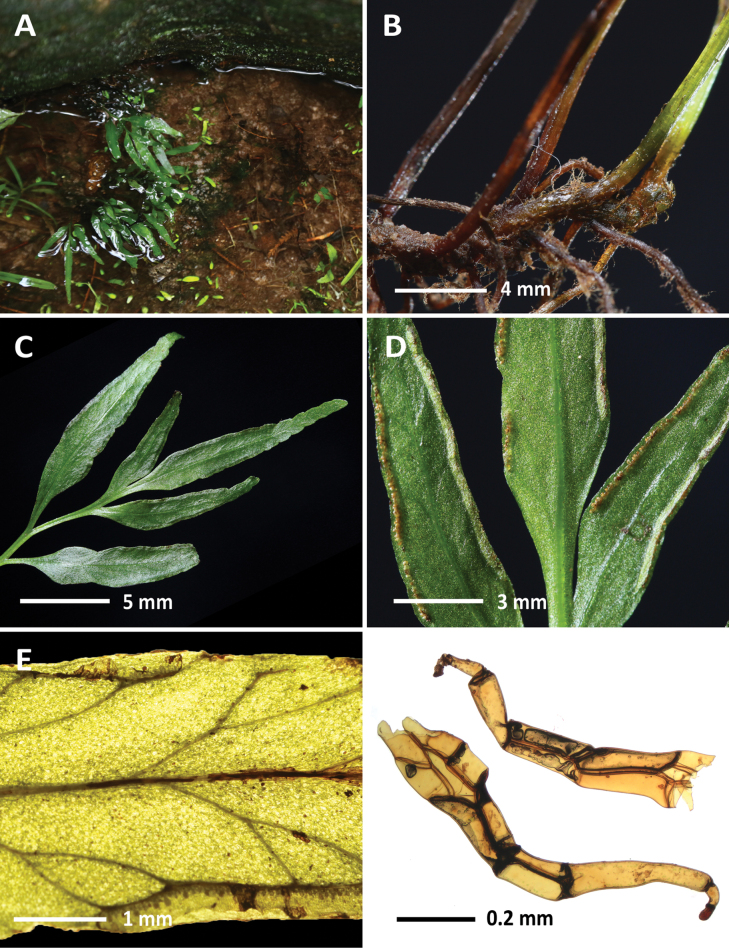
*Lindsaeakohkongensis* I.C.Hwang, M.O.Moon & B.Y.Sun (*Chen Wade5034*) **A** habit **B** rhizome **C** adaxial lamina **D** abaxial lamina **E** venation **F** rhizome scales. Photographed by C.-W. Chen.

**Figure 3.**
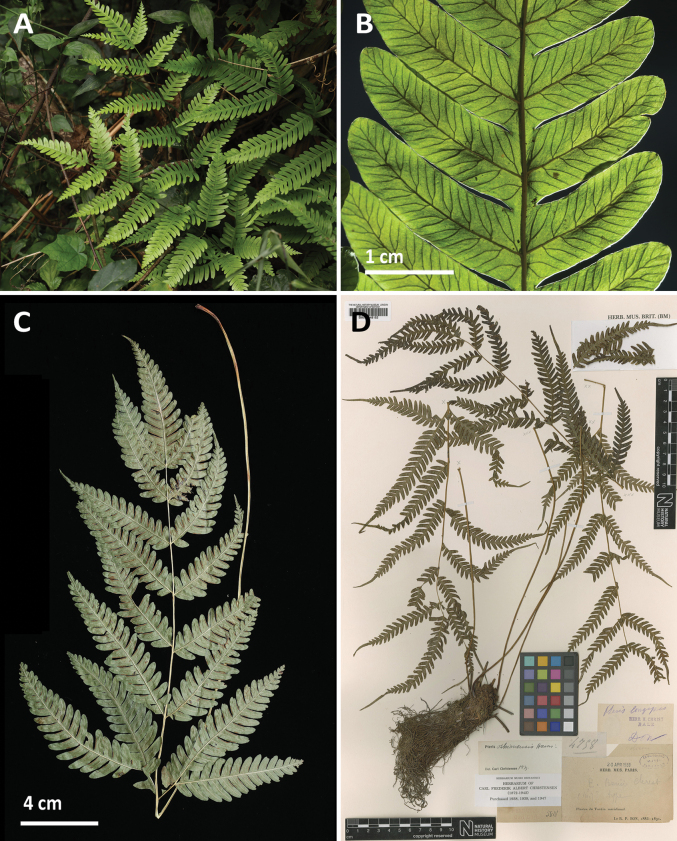
Comparison of *Pterispseudowulaiensis* C.M.Kuo (A, B, C, *Chao 3509 and 3517*) and *P.oshimensis* Hieron. (D, *Bon 4758*) **A** habit **B** lamina (backlight) **C** specimen **D** specimen, CC BY © The Trustees of the Natural History Museum, London. Photographed by C.-W. Chen and Y.-S. Chao.

This marks the third paper in our series on Vietnam’s pterido-flora (Chen et al. 2021; 2023). This series of studies aims to provide updated knowledge of the country’s pteridophyte flora, based on recent expeditions as well as the studies of herbarium specimens and relevant literature. Here, we reported three new additions to Vietnam’s flora: *Haplopterisyakushimensis* C.W.Chen & Ebihara (Fig. 1), *Lindsaeakohkongensis* I.C.Hwang, M.O.Moon & B.Y.Sun (Fig. 2), and *Pterispseudowulaiensis* Y.S.Chao (Fig. 3) based on our new collections.

**Figure 4.**
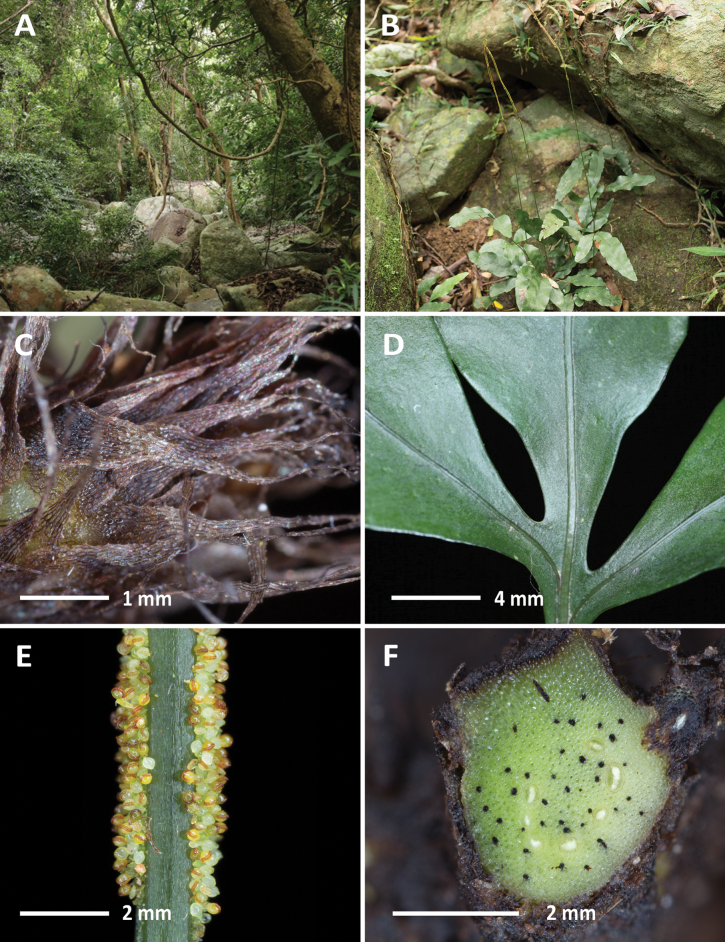
*Leptochiluspoilanei* (C.Chr. & Tardieu) Liang Zhang & Li Bing Zhang (*Chen Wade6804*) **A** habitat **B** habit **C** rhizome scales **D** sterile frond **E** fertile frond **F** cross section of rhizome. Photographed by C.-W. Chen.

Additionally, we performed phylogenetic analyses to elucidate the placements of *Lindsaeakohkongensis* (Fig. 2) and *Leptochiluspoilanei* (C.Chr. & Tardieu) Liang Zhang & Li Bing Zhang (Fig. 4). In 2018, we collected an unknown *Lindsaea* specimen from Phu Quoc Island of southern Vietnam. This specimen closely resembles *L.ensifolia* Sw. but being much smaller and with an unusual subaquatic habit. Specimens from Cambodia and Malaysia with the same morphology had recently been described as *Lindsaeakohkongensis* (Yun et al. 2023) but without molecular data. Here, we conducted a phylogenetic analysis to test whether *L.kohkongensis* is a distinct species or an eco-form of *L.ensifolia*.

*Leptochiluspoilanei* was described by Christensen and Tardieu-Blot (1939) under the genus *Colysis* (≡ *Leptochilus*), based on specimens collected from Annam, which is now part of central Vietnam. Following its initial description, the species remained largely unstudied until Zhang et al. (2018) reclassified it under *Leptochilus*. To our knowledge, this species is only known from its type collection, and our new collection (Fig. 4) from Nui Chua National Park represents the second collection of this species. It can be readily distinguished from other congeneric species in Vietnam by its strongly dimorphic fronds: fertile fronds are mostly linear and simple (rarely tripartite), while sterile ones are simple to pinnatifid.

Lastly, we presented new chromosome counts of Vietnamese fern species and explored the implications of our findings in the context of systematics. Chromosome numbers have long been recognized as important information in plant systematics (Rice et al. 2015). This significance is particularly pronounced for ferns, as they exhibit the highest frequency of polyploid speciation among vascular plants (Wood et al. 2009). Despite the importance of chromosome number data, research on Vietnamese ferns in this regard has been limited. To date, no systematic survey has been conducted. Instead, available data remains sporadic and dispersed throughout various articles, often in the context of new species’ descriptions (e.g., Chen et al. 2023), new species records (e.g., Ding et al. 2013), or cytological studies focused on specific taxa, including specimens collected from Vietnam (e.g., Nayak and Singh 1989). By reporting the chromosome numbers of Vietnamese fern species and demonstrating how these data can be integrated into systematic studies, we aim to raise awareness of chromosome studies among local botanists, thereby encouraging further related research in the future.

## ﻿Materials and methods

### ﻿Specimen identification and distribution

We conducted field expeditions in Bidoup Nui-Ba National Park, Cuc Phuong National Park, Phia Oac-Phia Den National Park, Phu Quoc National park, and Nui Chua National Park during 2018–2023. To ensure the correct identifications of our newly collected specimens, we compared them with herbarium specimens, relevant literature, including published papers, checklist, and flora from neighboring countries (see notes under each species), and original protologues and types through the Biodiversity Heritage Library (https://www.biodiversitylibrary.org/) and JSTOR Global Plants (https://plants.jstor.org/). To confirm the known distribution of each species, we searched the names against World Ferns (Hassler 1994–2024) and GBIF (https://www.gbif.org/) and manually examined the available specimen images.

### ﻿Cytological observations

To determine chromosome numbers, root tips were collected either from the field or transplanted plants in greenhouses. These root tips were treated with a 1:1 mixture of hydroxyquinoline and cycloheximide (Sigma-Aldrich, USA) for approximately 16 hours at 18 °C. Following this, the root tips were fixed in a 3:1 mixture of 95% ethanol and 45% acetic acid for about 12 hours at room temperature. Subsequently, the root tips were macerated using a 1:1 mixture of cellulase (Yakult, Japan) and pectolyase (Sigma-Aldrich, USA) for 1 hour at 37 °C. Finally, the treated root tips were squashed in 2% acetocarmine and observed under a microscope (Zeiss Axio Imager A1, Germany). To determine the ploidy of our observed chromosome numbers, we consulted two online databases, Chromosome Counts Database (CCDB, Rice et al. 2015) and Index to Plant Chromosome Numbers (IPCN, Goldblatt and Lowry 2011), and considered the lowest sporophytic counts known for a genus as the diploid.

Furthermore, we counted the spore numbers per sporangium and observed the spore shape regularity to determine the reproductive modes whenever possible. We also compared the spore sizes of *Leptochiluscantoniensis* (Baker) Ching and *L.poilanei* by measuring 30 spores from each species.

### ﻿Molecular phylogenetic analysis

To elucidate the phylogenetic placement of *Lindsaeakohkongensis* and *Leptochiluspoilanei*, we conducted two phylogenetic analyses each based on three chloroplast markers including *matK*, *trnH*-*psbA*, and *trnL*-*F* for *Lindsaea* and *rbcL*, *rps4-trnS*, and *trnL-F* for *Leptochilus*. These markers were chosen to integrate our newly generated sequences into previous studies (Lehtonen et al. 2010; Zhang et al. 2019; Chen et al. 2020a; Fujiwara et al. 2023). We extracted genomic DNA from fresh fronds using Qiagen DNeasy Plant Mini Kit (Hilden, Germany), following the manufacturer’s protocol. We conducted PCR to amplify the five DNA markers using the primers listed in Table 1.

**Table 1.** PCR Primers used in this study.

Region	Name	Sequence 5’ to 3’	Reference
*matK*	FERN matK fEDR	ATTCATTCRATRTTTTTATTTHTGGARGAYAGATT	Kuo et al. 2011
*matK*	DeLin matK rNRD	CTACGCAAYSCATCYCGATTT	Kuo et al. 2011
*rbcL*	1F	ATGTCACCACAAACAGAAAC	Fay et al. 1997
*rbcL*	1379R	TCACAAGCAGCAGCTAGTTCAGGACTC	Wolf et al. 1999
*rps4-trnS*	rps5	ATGTCCCGTTATCGAGGACCT	Nadot et al. 1995
*rps4-trnS*	trnSR	TACCGAGGGTTCGAATC	Smith and Cranfill 2002
*trnH-psbA*	trnH	CGCGCATGGTGGATTCACAATCC	Tate and Simpson 2003
*trnH-psbA*	psbA3’f	GTTATGCATGAACGTAATGCTC	Sang et al. 1997
*trnL-F*	FernL1Ir1	GGYAATCCTGAGCCAAATC	Li et al. 2009
*trnL-F*	F	ATTTGAACTGGTGACACGAG	Taberlet et al. 1991

For *Lindsaea*, we included the sequences of 26 specimens as listed in Appendix 1: Table A1. Among these, the sequences of 14 specimens were downloaded from GenBank. To test the monophyly of *L.ensifolia*, this sampling included 1) *L.ensifolia* from a broad geographic range, including Bangladesh, Brunei, Cambodia, India, Malaysia, Nepal, Solomon Islands, Thailand, and Vietnam; and 2) closely related species identified by Lehtonen et al. (2010). For *Leptochilus*, in addition to our newly sequenced *L.poilanei*, we download the sequences of another 21 specimens from GenBank. In total, 22 specimens representing 19 species were included in the phylogenetic analysis as listed in Appendix 1: Table A2. This sampling covered all the major clades found in previous studies (Zhang et al. 2019; Chen et al. 2020a).

For our newly generated sequences, we first manually inspected raw reads, removing any ambiguous bases using BioEdit (Hall 1999). Subsequently, we aligned the sequences of each marker using MUSCLE (Edgar 2004) with default settings. These individual marker alignments were then concatenated into a single alignment, because chloroplast genome is non-recombining and therefore has a single evolutionary history. We then conducted maximum likelihood (ML) analyses using IQTREE (Minh et al. 2020) each with five partitions (three codon positions for *matK* and *rbcL*, and *rps4-trnS*, *trnH*-*psbA* and *trnL-F*, each treated as a single partition for simplification although they contain partly coding regions). The best-fit model for each partition and the best-fit partition scheme were determined by ModelFinder (Lanfear et al. 2012; Kalyaanamoorthy et al. 2017) as implemented in IQTREE (Minh et al. 2020). To assess branch support, we performed 1000 ultrafast bootstrap replicates using UFBoot (Hoang et al. 2018). The two concatenated alignments and the resulting phylogenetic trees are available on the Dryad Digital Repository (Chen et al. 2024).

## ﻿Results

### ﻿Phylogenetic placement of *Lindsaeakohkongensis*

**Figure 5.**
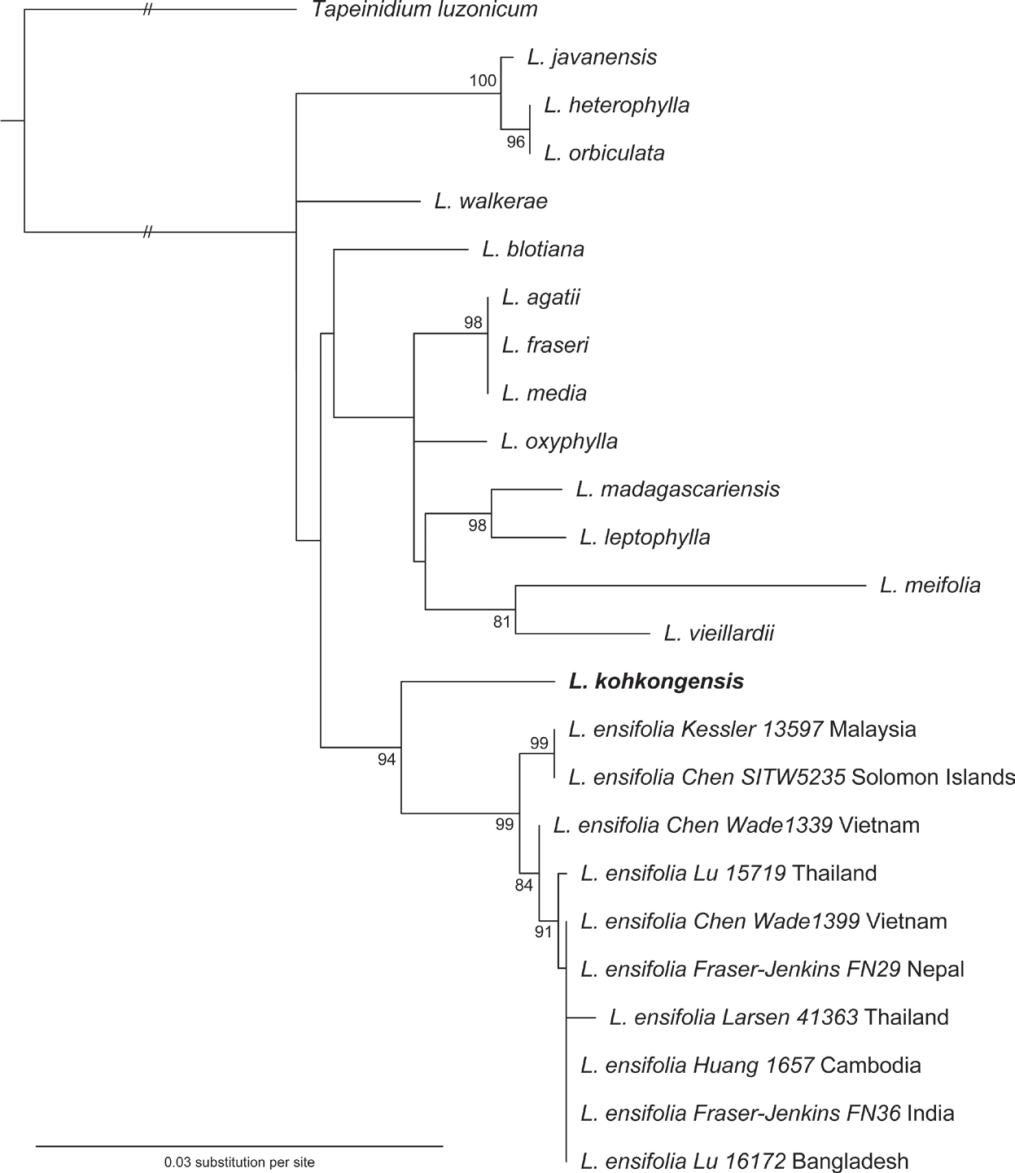
Phylogram of *Lindsaea* reconstructed from the maximum likelihood analysis of the concatenated plastid dataset (*matK*, *trnH*-*psbA*, and *trnL-F*). The branch length to the outgroup *Tapeinidium* is not to scale for better visualization. Support values below 80 are not shown on the nodes. *L.kohkongensis* is indicated in bold.

The concatenated three-marker alignment contains 1832 bp including 85 parsimony informative sites and 39.2% missing data (17,973 bp). ModelFinder merged all the five partitions into one alignment with the best-fit model identified as K3Pu+F+R2. The phylogram generated by the ML analysis based on the concatenated alignment is shown in Fig. 5. The inferred species relationships closely align with the phylogeny reconstructed by Lehtonen et al. (2010) although the nodes are poorly supported in general. However, *Lindsaeakohkongensis* is strongly supported as the sister group to a clade comprising all the *L.ensifolia* specimens included in this analysis.

### ﻿Phylogenetic placement of *Leptochiluspoilanei*

**Figure 6.**
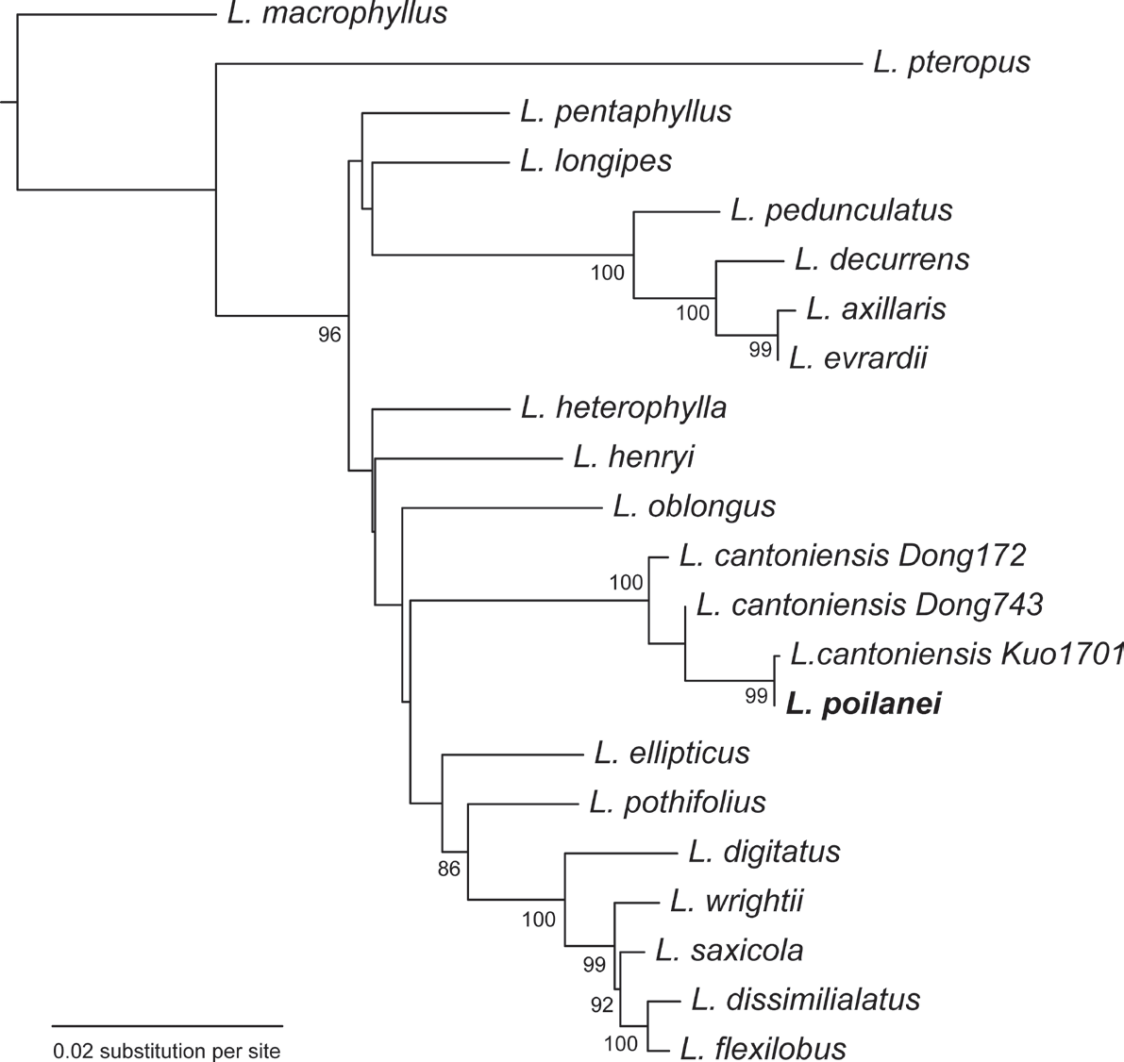
Phylogram of *Leptochilus* reconstructed from the maximum likelihood analysis of the concatenated plastid dataset (*rbcL*, *rps4-trnS*, and *trnL-F*). Support values below 80 are not shown on the nodes. *L.poilanei* is indicated in bold.

The concatenated three-marker alignment contains 3331 bp including 248 parsimony informative sites and 17.8% missing data (13077 bp). ModelFinder subset the alignment into two partitions, the first including first and third codon position of *rbcL*, and the second including the second codon position of *rbcL* along with *rps4-trnS* and *trnL-F*. The best-fit models determined for the two partitions were TNe+I+G4 and K3Pu+F+R2, respectively. The phylogram generated by the ML analysis based on the concatenated alignment is shown in Fig. 6. The inferred species relationships are similar to previous studies (Zhang et al. 2019; Chen et al. 2020a) as expected, although poorly supported in general. *Leptochiluspoilanei* is nested in a highly supported clade containing three specimens of *L.cantoniensis*.

Both *L.cantoniensis* (based on *Hsu 3399*) and *L.poilanei* (based on *Chen Wade6804*) produce 64 spores in each sporangium with normal morphology. The spore size of *L.cantoniensis* and *L.poilanei* is 44.3 ± 3.4 and 63.0 ± 4.9 µm, respectively. The spores of *L.poilanei* are significantly larger than those of *L.cantoniensis* (t-test, *p* < 0.001).

### ﻿Chromosome number of 20 species

**Figure 7.**
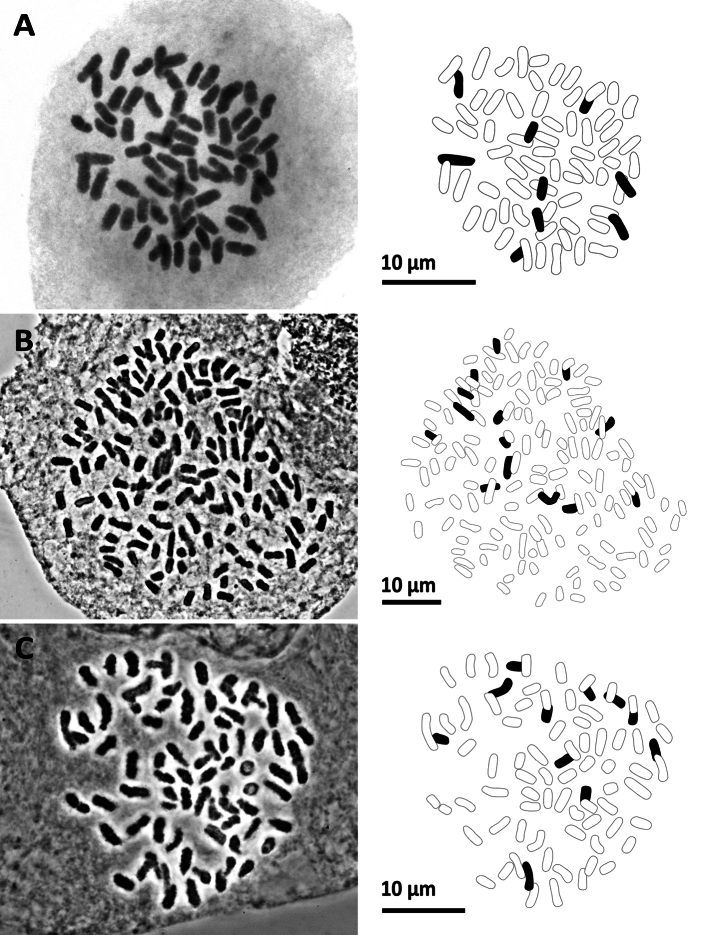
Mitotic chromosomes with explanatory illustrations **A***Aspleniumnormale* (2*n* = 72) **B***Aspleniumtenerum* (2*n* = 144) **C***Ctenitiseatonii* (2*n* = 82).

**Figure 8.**
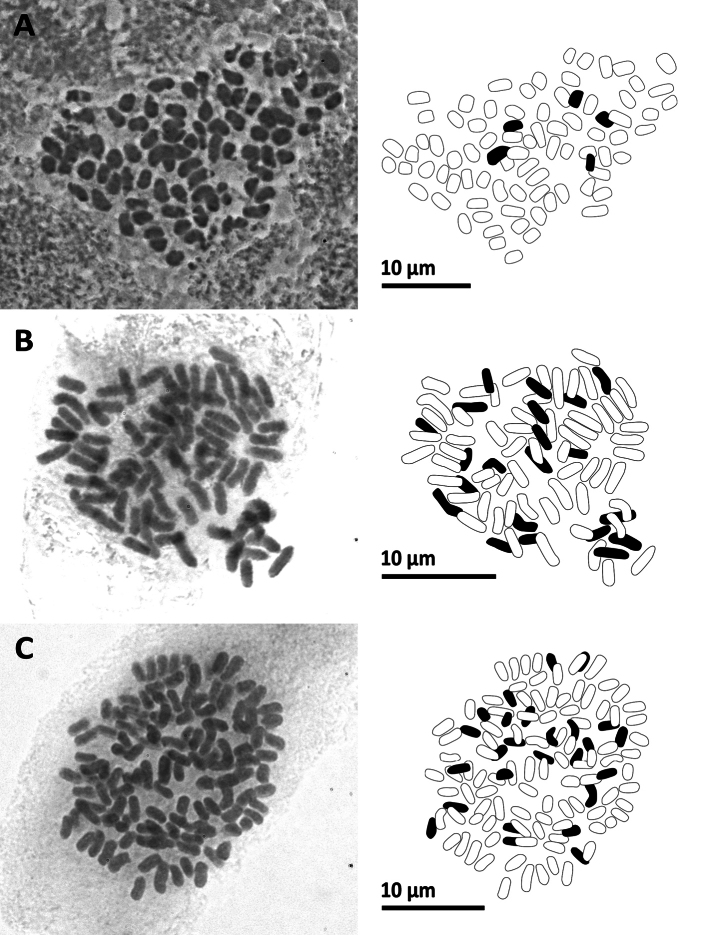
Mitotic chromosomes with explanatory illustrations **A***Didymochlaenatruncatula* (2*n* = 82) **B***Diplaziumdoederleinii* (2*n* = 82) **C***Diplaziumdonianum* (2*n* = 123).

**Figure 9.**
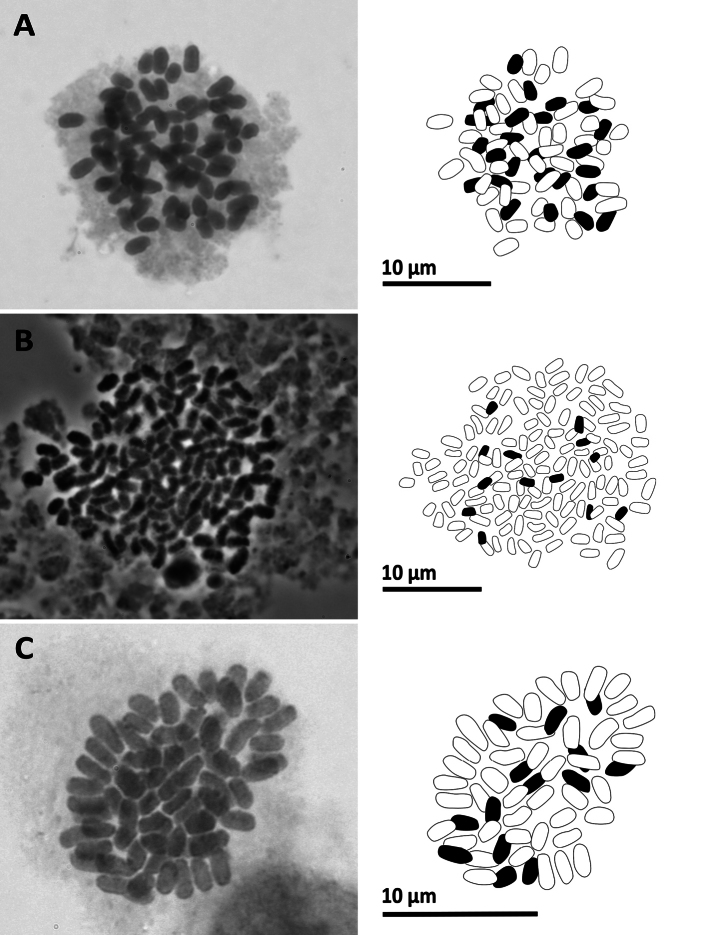
Mitotic chromosomes with explanatory illustrations **A***Grypothrixsimplex* (2*n* = 72) **B***Gymnosphaerasalletii* (2*n* = 138) **C***Lepisorusspicatus* (2*n* = 70).

**Figure 10.**
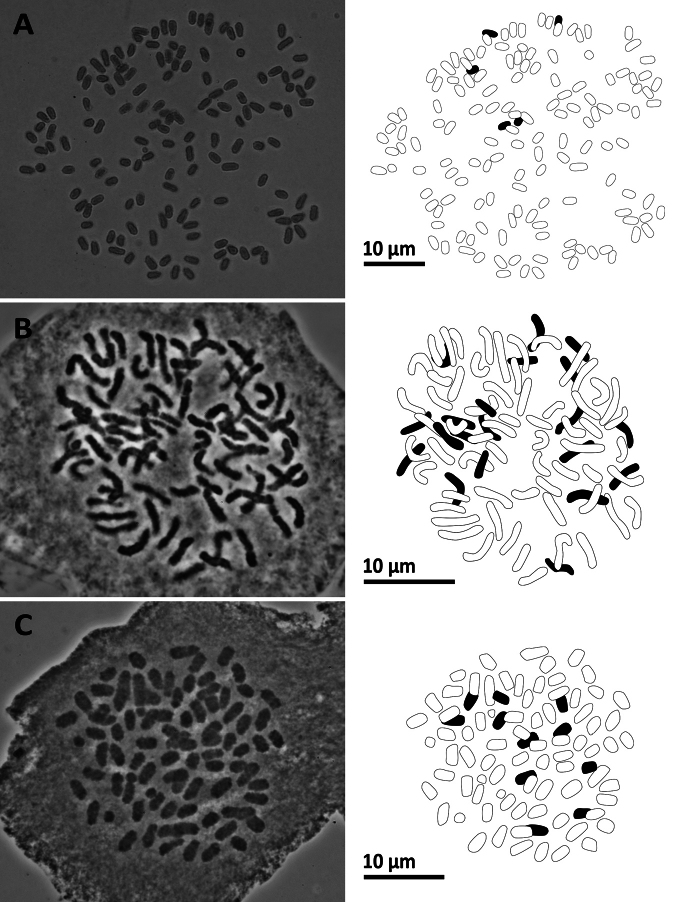
Mitotic chromosomes with explanatory illustrations **A***Leptochiluspoilanei* (2*n* = 144) **B***Pleocnemiawinitii* (2*n* = 82) **C***Polystichumbiaristatum* (2*n* = 82).

**Figure 11.**
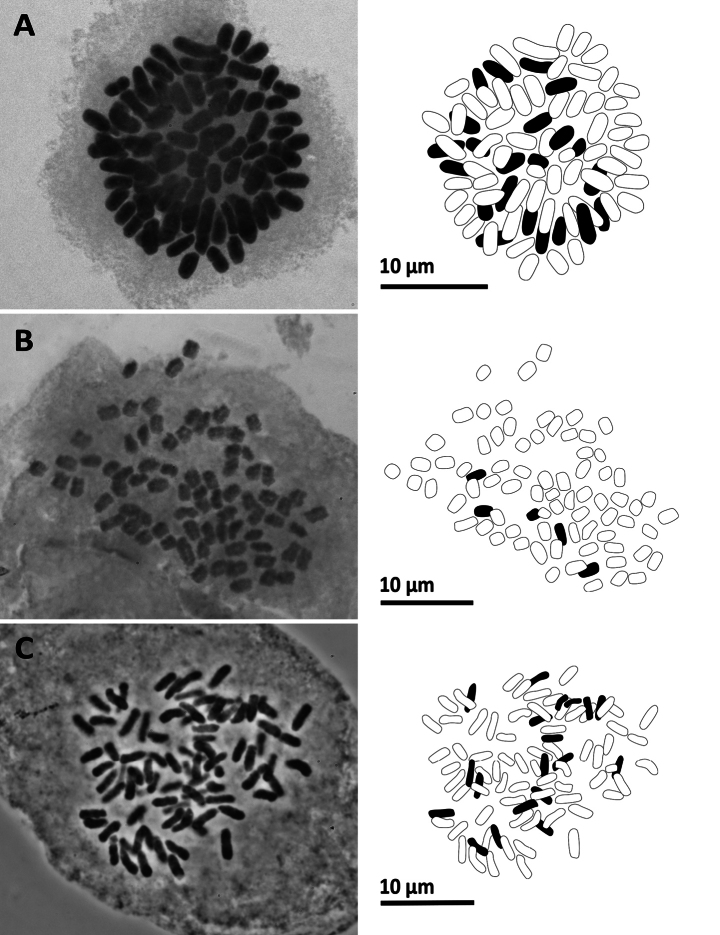
Mitotic chromosomes with explanatory illustrations **A***Pteridryscostularis* (2*n* = 82) **B***Pteriscadieri* (2*n* = 87) **C***Pteriscadieri* (2*n* = 87).

**Figure 12.**
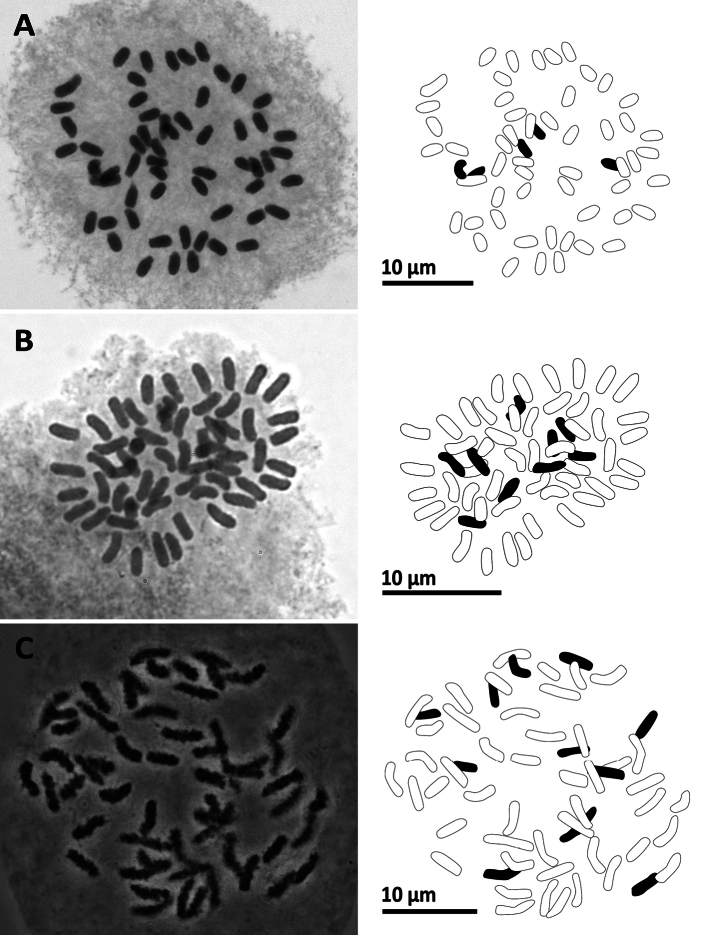
Mitotic chromosomes with explanatory illustrations **A***Pterisesquirolii* (2*n* = 58) **B***Pterislatipinna* (2*n* = 58) **C***Pterispseudowulaiensis* (2*n* = 58).

**Figure 13.**
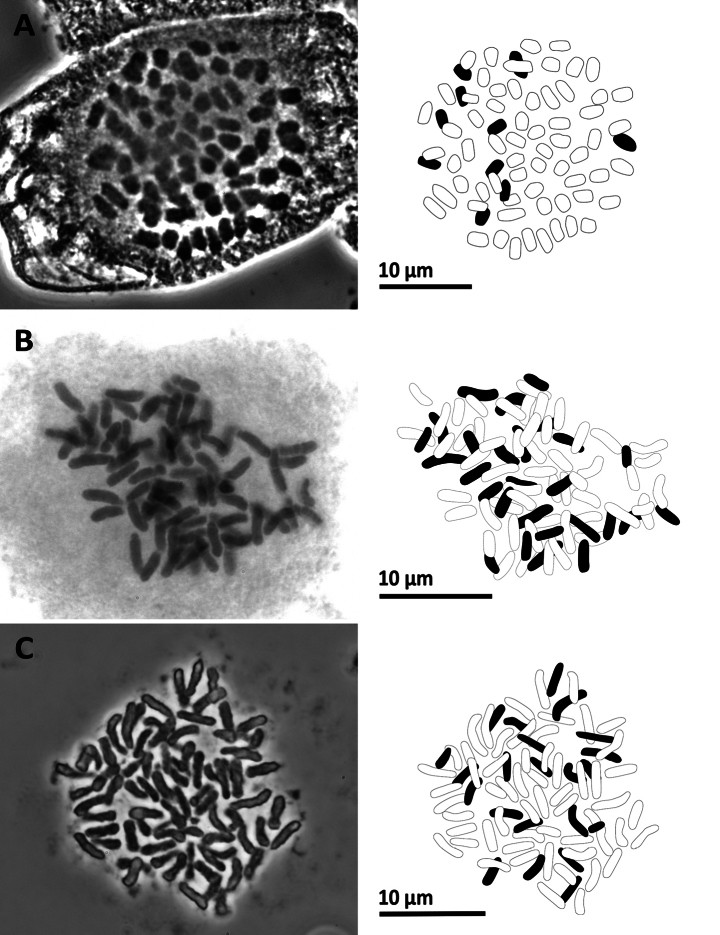
Mitotic chromosomes with explanatory illustrations **A***Pyrrosiaeberhardtii* (2*n* = 74) **B***Tectariaharlandii* (2*n* = 80) **C***Tectariasetulosa* (2*n* = 80).

We successfully counted the chromosome numbers of 20 species, each determined by multiple cells. Their chromosome numbers and ploidy levels inferred from the known lowest base number are shown in Table 2. Figs 7–13 illustrated representative cells for all the 20 species. Among these, chromosome numbers were recorded for the first time for seven species: *Gymnosphaerasalletii* (Tardieu & C.Chr.) S.Y.Dong, *Lepisorusspicatus* (L.f.) Li Wang, *Leptochiluspoilanei*, *Pteridryscostularis* Li Bing Zhang, Liang Zhang, N.T.Lu & X.M.Zhou, *Pterislatipinna* Y.S.Chao & W.L.Chiou, *Pyrrosiaeberhardtii* (Christ) Ching, and *Tectariasetulosa* (Baker) Holttum. Furthermore, we reported the new cytotypes for three species: *Diplaziumdoederleinii* (Luerss.) Makino, *Pterisesquirolii* H.Christ, and *Tectariaharlandii* (Hook.) C.M.Kuo.

**Table 2.** Plant materials used in this study for chromosome counting, their inferred ploidy, spores number in each sporangium (s/s), and corresponding figures. Asterisks “*” indicate newly reported species. Hash marks “#” indicate newly reported cytotypes. Em-dashes “—” indicate missing data.

Taxa	Voucher	Chromosome no.	Ploidy	s/s	Figure
*Aspleniumnormale* D.Don	*Chen Wade5121*	2*n* = 72	2x	64	7A
*Aspleniumtenerum* G.Forst.	*Chen Wade5342*	2*n* = 144	4x	64	7B
*Ctenitiseatonii* (Baker) Ching	*Chen Wade6815*	2*n* = 82	2x	—	7C
*Didymochlaenatruncatula* (Sw.) J.Sm	*Chen Wade5817*	2*n* = 82	2x	64	8A
*Diplaziumdoederleinii* (Luerss.) Makino#	*Chao 3524*	2*n* = 82	2x	64	8B
*Diplaziumdonianum* (Mett.) Tardieu	*Chen Wade6853*	2*n* = 123	3x	—	8C
*Grypothrixsimplex* (Hook.) S.E.Fawc. & A.R.Sm	*Chen Wade6879*	2*n* = 72	2x	64	9A
*Gymnosphaerasalletii* (Tardieu & C.Chr.) S.Y.Dong*	*Chen Wade6586*	2*n* = 138	2x	64	9B
*Lepisorusspicatus* (L.f.) Li Wang*	*Chen Wade6598*	2*n* = 70	2x	—	9C
*Leptochiluspoilanei* (C.Chr. & Tardieu) Liang Zhang & Li Bing Zhang*	*Chen Wade6804*	2*n* = 144	4x	64	10A
*Pleocnemiawinitii* Holttum	*Chao 3534*	2*n* = 82	2x	64	10B
*Polystichumbiaristatum* (Blume) T.Moore	*Chen Wade5070*	2*n* = 82	2x	64	10C
*Pteridryscostularis* Li Bing Zhang, Liang Zhang, N.T.Lu & X.M.Zhou*	*Chen Wade6819*	2*n* = 82	2x	—	11A
*Pteriscadieri* H.Christ	*Chen Wade5737*, *Chao 3501*	2*n* = 87	3x	32	11B, 11C
*Pterisesquirolii* H.Christ#	*Chao 3511*	2*n* = 58	2x	64	12A
*Pterislatipinna* Y.S.Chao & W.L.Chiou*	*Chao 3526*	2*n* = 58	2x	32	12B
*Pterispseudowulaiensis* C.M.Kuo	*Chao 3515*	2*n* = 58	2x	32	12C
*Pyrrosiaeberhardtii* (Christ) Ching*	*Chen Wade5607*	2*n* = 74	2x	—	13A
*Tectariaharlandii* (Hook.) C.M.Kuo#	*Chen Wade6874*	2*n* = 80	2x	—	13B
*Tectariasetulosa* (Baker) Holttum *	*Chen Wade6852*	2*n* = 80	2x	64	13C

## ﻿Discussion

### ﻿Systematic uniqueness of *Lindsaeakohkongensis*

When we collected this species from Phu Quoc island in 2018, we were hesitant to describe it. Morphologically, it closely resembles *L.ensifolia*, leading us to question if it might be an eco-form of the latter. In this study, we conducted a phylogenetic analysis based on three chloroplast regions, sampling *L.ensifolia* from a wide geographic distribution throughout Asia and the Pacific Islands. The results indicate that *L.kohkongensis* is sister to *L.ensifolia* rather than being embedded within it (Fig. 6). On one hand, this suggests that *L.kohkongensis* can be recognized as a distinct species based on the principle of monophyly. On the other hand, it could also be classified within a broadly circumscribed *L.ensifolia*. To our knowledge, *L.ensifolia* typically grows terrestrially in a diverse habitat from swampy forests, dipterocarp forests, to exposed grasslands (Kramer 1971; personal observation). In contrast, *L.kohkongensis* is exclusively found in shaded valleys as lithophytes and is frequently submerged in water (Yun et al. 2023; personal observation). This distinction suggests an ecological speciation event that needs to be tested in the future.

While identifying our specimen from Phu Quoc island, we came across a rheophyte form of *L.ensifolia* described as L.ensifoliavar.rheophila K.Iwats from Sumatra (Iwatsuki 1977). After examining the type materials deposited in KYO, we concluded that this is the same species as *L.kohkongensis* and should be treated as a synonym of it. As noted by Kramer (1967), *L.ensifolia* is one of the most widespread and variable species of the genus. Apart from var. rheophila, few other subspecies or varieties have been published, such as subsp. coriacea (Alderw.) K.U.Kramer from Bornean swamp forests. Furthermore, ploidy variations (diploid, triploid, and tetraploid) and different reproductive modes (sexual and apomixis) have been observed in *L.ensifolia* (e.g., Lin et al. 1996). Although we here tentatively recognize *L.kohkongensis* as a distinct species, the variability observed in *L.ensifolia* underscores the need for a systematic study of this complex species.

### ﻿Hybrid origin hypothesis of *Leptochiluspoilanei*

**Figure 14.**
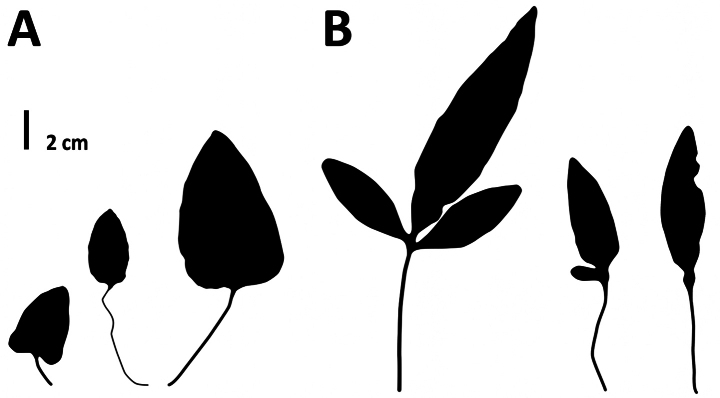
Comparison of the sterile fronds between *Leptochiluscantoniensis* and *L.poilanei***A***L.cantoniensis*, based on *Kuo 1701* (TAIF [509328], left), *Cadière 158* (MICH [1191289], middle), and *s.c.*, *s.n.* (K [000959730], right) **B***L.poilanei*, based on *Poilane 5373* (BM [000036782], all the three fronds).

Our phylogenetic analysis based on three plastid markers unambiguously resolves *L.poilanei* within a clade that includes three specimens of *L.cantoniensis* (Fig. 6). This result is unexpected, considering the clear morphological distinctions between these two species. Specifically, fully developed sterile fronds of *L.poilanei* are pinnatifid, while those of *L.cantoniensis* remain consistently simple (Fig. 14).

Our examination of the reproductive mode indicates that both species undergo sexual reproduction, as evidenced by the production of 64 well-formed spores in each sporangium. The mitotic chromosome count for *L.poilanei* is 2*n* = 144 (Fig. 10A), suggesting it is a tetraploid, given the base number of the genus is *x* = 36 (Rice et al. 2015). Although the chromosome number of *L.cantoniensis* is presently unknown, its spores are significantly smaller than those of *L.poilanei* (44.3 ± 3.4 vs. 63.0 ± 4.9 µm). Considering the widely found correlation between ploidy and spore size in ferns (Barrington et al. 1986), we propose that *L.cantoniensis* is likely a diploid species.

Building on the evidence from our study, we propose that *L.poilanei* has a hybrid origin. We suggest that *L.poilanei* originated from hybridization between *L.cantoniensis* (as the maternal parent, considering chloroplast inheritance in ferns, e.g., Gastony and Yatskievych 1992) and an unidentified diploid species, followed by polyploidization. The close similarity in chloroplast DNA markers between *L.cantoniensis* and *L.poilanei* suggests a relatively recent hybridization event. This hypothesis gains further support from the overlapping distribution of *L.cantoniensis* and *L.poilanei* in central Vietnam. While the identity of the other parent remains elusive, we anticipate it is likely a species with pinnatifid fronds, given that *L.poilanei* exhibits this characteristic. Future studies employing bi-parentally inherited nuclear markers and comprehensive sampling of the genus in Vietnam are essential to test our hypothesis.

### ﻿New findings of chromosome number

In the following paragraphs, we discussed the systematic implications of our new finding (i.e., new species counts or new cytotypes) species by species, following alphabetical order.

#### ﻿*Diplaziumdoederleinii* (Luerss.) Makino—2*n* = 82, sexual diploid, Fig. 8B

Previous studies reported triploid (2*n* = 123) and tetraploid (2*n* = 164) specimens from Japan (Takamiya et al. 2001). Our discovery of a diploid specimen from Vietnam provides further support for the long-observed cytogeographic pattern: diploids are typically found in warmer tropics, whereas polyploids are prevalent in cooler temperate regions (e.g. Chang et al. 2013). As demonstrated by Takamiya et al. (2001), there is a little genetic divergence between triploids and tetraploids. Future studies could incorporate the newly discovered diploid into genetic analyses to further investigate the origins of polyploids.

#### ﻿*Gymnosphaerasalletii* (Tardieu & C.Chr.) S.Y.Dong—2*n* = 138, sexual diploid, Fig. 9B

The first chromosome count reported for the species. This Vietnam endemic species was recently reevaluated by Li et al. (2024), who described two new species previously been misidentified as *G.salletii*. *Gymnosphaera* is predominantly a paleotropical genus, comprising approximately 46 species (Dong and Zuo 2018). All four previously reported species with chromosome counts in the genus are diploid, namely *G.capensis* (L.f.) S.Y.Dong, *G.gigantea* (Wall. ex Hook.) S.Y.Dong, *G.khasyana* (T.Moore ex Kuhn) Ching, and *G.podophylla* (Hook.) Copel. (Manton and Sledge 1954; Löve et al. 1977; Kato 1999). Notably, there is a correlation between geographic distribution and the basic chromosome number: all Asian species exhibit *x* = 69, while African species have *x* = 70.

#### ﻿*Lepisorusspicatus* (L.f.) Li Wang—2*n* = 70, diploid, Fig. 9C

The first chromosome count reported for the species. Found across the paleotropics, this species has often been confused with *L.mucronatus* (Fée) Li Wang. Following Hovenkamp (1998), we identify the species by the presence of entire rhizome scales with hyaline margins. Following Zhao et al. (2020), this species belongs to sectiob Belvisia (Mirb.) C.F. Zhao, R.Wei & X.C. Zhang, a small clade comprising eight species (Hovenkamp and Franken 1993). *Lepisorusmucronatus* is the only other species in this section with known chromosome numbers and has been determined as tetraploid (2*n* = 140) from Australia (Tindale and Roy 2002).

#### ﻿*Leptochiluspoilanei* (C.Chr. & Tardieu) Liang Zhang & Li Bing Zhang—2*n* = 144, sexual tetraploid, Fig. 10A

As discussed in previous sections, we hypothesize that this Vietnam endemic species originated from a hybridization between *L.cantoniensis* and an unidentified diploid species, followed by polyploidization. *Leptochilus* is predominantly a tropical Asian genus with approximately 51 species (Zhang et al. 2024). Prior to this study, chromosome numbers were known for only four species including: *L.axillaris* (Cav.) Kaulf., *L.decurrens* Blume, *L.ellipticus* (Thunb.) Noot., and *L.pothifolius* (Buch.-Ham. ex D.Don) Fraser-Jenk. (e.g., Mitui 1968; Löve et al. 1977; Manickam and Irudayaraj 1989). Among these four species, *L.decurrens* is the only one reported to exist in both diploids and tetraploids, while the other three species are known only as diploids.

#### ﻿*Pteridryscostularis* Li Bing Zhang, Liang Zhang, N.T.Lu & X.M.Zhou—2*n* = 82, diploid, Fig. 11A

The first chromosome count reported for the species. *Pteridrys* is a tropical Asian genus with ca. 22 species, and 11 of them are recorded in Vietnam (Zhou and Zhang 2019). So far, all the four species (the other three are *P.australis* Ching ex C.Chr. & Ching, *P.cnemidaria* (Christ) C.Chr.et.Ching, and *P.syrmatica* (Willd.) C.Chr. & Ching) with reported chromosome numbers are diploids (Manton 1954; Tsai and Shieh 1985; Bidin and Go 1995).

#### ﻿*Pterisesquirolii* H.Christ—*2n* = *58*, sexual diploid, Fig. 12A

This species is reported from southern China, Taiwan, and Vietnam (Chao 2019). Prior to this study, the only reported chromosome count was 2*n* = ca. 90 (presumably an apogamous triploid due to the count of 32 spores per sporangium) from southern China (Lin et al. 2002). Although Chao (2019) found very little genetic divergence among populations from China and Taiwan, it may be worthwhile to include the Vietnamese population in future analyses, given their different ploidy level and reproductive mode. This is particularly important considering the prevalence of reticulate evolution observed in the genus (e.g., Chao et al. 2012, 2022).

#### ﻿*Pterislatipinna* Y.S.Chao & W.L.Chiou—*2n* = *58*, apogamous diploid, Fig. 12B

This is the first chromosome count reported for this species. This species was firstly described from Taiwan (Chao et al. 2017) and later reported from China and Vietnam (Chen et al. 2020b). Our chromosome counts support the hypothesis proposed by Chao et al. (2022) that *P.latipinna* is a diploid, as indicated by flow cytometry. Our spore number counts also confirm that *P.latipinna* is reproduced through apogamous reproduction, which is common for the genus (Walker 1962).

#### ﻿*Pyrrosiaeberhardtii* (Christ) Ching—2*n* = 74, diploid, Fig. 13A

The first chromosome count reported for the species. This species is recorded in southern China, Thailand, and Vietnam, with its type locality in Vietnam. It has sometimes been grouped under a broadly defined *P.lingua* (Thunb.) Farw. alongside other closely related species such as *P.oblonga* Ching and *P.heteractis* (Mett.) Ching. (Hovenkamp 1986). This broader classification of *P.lingua* is further supported by Zhou et al. (2017), showing a close relationship among these species. The chromosome numbers of *P.lingua* have been reported from China and Japan, and both are diploids (e.g., Takei 1969; Kato 1999).

#### ﻿*Tectariaharlandii* (Hook.) C.M.Kuo—2*n* = 80, diploid, Fig. 13B

This species has been documented in the Ryukyu Islands, southern China, northern Vietnam, and Taiwan. Tetraploid populations (*n* = 80) have been reported in the Ryukyu Islands (as *Hemigrammadecurrens* (Hook.) Copel., Mitui 1976) and Taiwan (Tsai and Shieh 1985). In our study, we provide the first record of a diploid form Vietnam. Notably, this species has been proposed as the maternal parent of the triploid (*2n* = 120) sterile hybrid species *T.×hongkongensis* (Zhao and Dong 2016), with *T.zeilanica* (Houtt.) Sledge as the paternal parent. It is reasonable to assume that *T.×hongkongensis* originated from the hybridization between diploid and tetraploid parents. Given that both diploids and tetraploids are now confirmed in these two parental species (Manton and Sledge 1954; Tsai and Shieh 1985), it would be interesting to investigate whether there is a bias in the direction of hybridization based on ploidy level.

#### ﻿*Tectariasetulosa* (Baker) Holttum—2*n* = 80, sexual diploid, Fig. 13C

The first chromosome count reported for the species. Initially described from Ba Vi mountain range in northern Vietnam, this species has since been documented in southern China, Indochina, and Peninsular Malaysia. Additionally, a variety *raciborskii* (Alderw.) Holttum has been further identified extending to Java without known ploidy.

### ﻿New records from Vietnam

#### ﻿ Haplopteris yakushimensis

Taxon classificationPlantaePolypodialesPteridaceae

C.W.Chen & Ebihara, Phytotaxa 156(4): 232. 2014.

9C99E1EE-788C-56A3-AB12-8D65D377F4FF

Fig. 1

##### Type.

Japan. • Kagoshima Pref., Yakushima Island, Nakabase River, 20 Aug 1982, *Nakaike s.n.* (holotype TNS [VS-456666!]).

##### Distribution and ecology.

This species was previously recorded only in Japan and Taiwan (Chen et al. 2014, TPG 2019). In Vietnam, *H.yakushimensis* is found in damp evergreen broadleaf forests at elevations of 1200–1360 m, where it grows on rocks with thick compost near streams.

##### Specimens examined.

Vietnam. • Cao Bang Province: Phia-Oac Phia-Den National Park, 6 Dec 2013, *Zhang et al. 6755* (MO, TAIF [499240!], VNMN). Phia-Oac Phia-Den National Park, Nguyen Binh District, Thanh Cong Ward, 22.589539°N, 105.880403°E, 1353 m, 8 Nov 2023, *Chen Wade6952* (TAIF!, VNMN!). • Ha Giang Province: Vi Xuyen District, Cao Bo Ward, 22.767500°N, 104.880394°E, 1200–1360 m, 12 Sep 2000, *Harder et al. 5514* (UC [1763099!]).

##### Note.

*Haplopteris*, a fern genus comprising approximately 40 species, is primarily found in tropical Africa, Asia, and the Pacific Islands (Schuettpelz et al. 2016). In Vietnam, eight *Haplopteris* species were previously recorded: *H.angustifolia* (Blume) E.H.Crane, *H.doniana* (Mett. ex Hieron.) E.H.Crane, *H.elongata* (Sw.) E.H.Crane, *H.ensata* (Christ) C.W.Chen & S.Linds., *H.ensiformis* (Sw.) E.H.Crane, *H.flexuosa* (Fée) E.H.Crane, *H.hainanensis* (C.Chr. ex Ching) E.H.Crane, and *H.sikkimensis* (Kuhn) E.H.Crane (Chen et al. 2023). *Haplopterisyakushimensis* can be distinguished from these species by having fronds broader than 1 cm wide, costae that are grooved on the adaxial side and raised on the abaxial side, and the submarginal (ca. 1–2 mm away from the margins) sori lines.

Recent studies have identified two genetically distinct yet morphologically indistinguishable lineages in *H.yakushimensis* (Kuo et al. 2017; TPG 2019). With the discovery of new populations in Vietnam, we are currently working to clarify these lineages using an integrated approach that includes reproductive biology, genome size estimation, and nuclear markers. The results of this research will be published in a separate paper.

#### ﻿ Lindsaea kohkongensis

Taxon classificationPlantaePolypodialesLindsaeaceae

I.C.Hwang, M.O.Moon & B.Y.Sun, Korean J. Pl. Taxon. 53(4): 289. 2023.

4A3F447C-5DB1-5B35-8B5F-CB3066F0443B

Fig. 2

##### Type.

Cambodia • Koh Kong, Thma Bang District, near Chamnar village, 22 Dec 2013, *Sun et al. C5520* (holotype HIBR; isotypes HIBR, KB).

##### Distribution and ecology.

This species was recently described from Cambodia and Malaysia. In Vietnam, it grows along a valley in damp tropical forests as lithophytes and is frequently submerged in water.

##### Specimens examined.

Vietnam. • Kien Giang Province: Phu Quoc district, Phu Quoc National Park, 6 Mar 2018, *Chen Wade5034* (TAIF [514046!]).

##### Note.

Morphologically, this species closely resembles *Lindsaeaensifolia*. Although *L.kohkongensis* is usually a much smaller species than *L.ensifolia*, we did not identify any qualitative trait to distinguish them. Currently, subaquatic habitat preference appears to be the most reliable characteristic for differentiating this species from *L.ensifolia*. Yun et al. (2023) described *L.kohkongensis* as having free venation, in contrast to the anastomosing venation found in *L.ensifolia*. However, this is not true. As demonstrated in Fig. 3G of their paper and Fig. 2F of the current study, the veins of *L.kohkongensis* are connected near the lamina margin, forming areoles in fertile fronds.

#### ﻿ Pteris pseudowulaiensis

Taxon classificationPlantaePolypodialesPteridaceae

Y.S.Chao, Taiwania 66(3): 314. 2021.

FFBC7C92-BCCB-5197-9143-06B00505EE9C

Fig. 3

##### Type.

Taiwan • New Taipei, Mt. Pataoerh, 600–700 m, 29 Apr 2016, *Hsu 8437* (holotype TAIF [497137!]; isotype TAIF [497138!]).

##### Distribution and ecology.

This species, recently described from Taiwan, was initially reported from southern China and Taiwan (Chao et al. 2021). In Vietnam, *P.pseudowulaiensis* was discovered in relatively dry evergreen broadleaf forests at lowland areas of Cuc Phuong National Park.

##### Specimens examined.

Vietnam • Ninh Binh Province: Cuc Phuong National Park, Muong Khu trail, *Y.-S. Chao 3509*, *3515*, *3517*, *3531* (TAIF!, VNMN!).

##### Note.

This species belongs to the *Pterisfauriei* Hieron. complex, a taxonomically challenging group due to its reticulate evolution involving hybridization, polyploidization, and apomixis (Chao et al. 2022). In Vietnam, five species from this complex have been recorded, namely *P.arisanensis* Tagawa (Chao et al. 2022), *P.kawabatae* Sa.Kurata (Chao et al. 2021), *P.latipinna* Y.S.Chao & W.L.Chiou (Chen et al. 2020b), *P.oshimensis* Hieron. (Liao et al. 2013), and *P.pseudowulaiensis*. Morphologically, *P.pseudowulaiensis* closely resembles *P.oshimensis* but differs by having broader pinnae, measuring 2–3.5 cm compared to less than 2 cm in *P.oshimensis*. According to Chao et al. (2022), *P.pseudowulaiensis* is an apogamous diploid originating from hybridization of *P.wulaiensis* C.M.Kuo (a species reported from Japan and Taiwan) and another unknown species.

## Supplementary Material

XML Treatment for Haplopteris yakushimensis

XML Treatment for Lindsaea kohkongensis

XML Treatment for Pteris pseudowulaiensis

## ﻿Additional information

### Author ORCIDs

Cheng-Wei Chen https://orcid.org/0000-0001-9709-6739

Van Truong Do https://orcid.org/0000-0002-0585-5513

Hong Truong Luu https://orcid.org/0000-0002-7036-7081

Yi-Shan Chao https://orcid.org/0000-0001-7433-5987

Kuo-Fang Chung https://orcid.org/0000-0003-3628-2567

### Data availability

All of the data that support the findings of this study are available in the main text.

## ﻿Appendix 1

**Table A1.** Specimens of *Lindsaea* used for molecular phylogenetic analysis in this study. New sequences generated in this study are indicated in bold. En-dashes “–” indicate missing data.

Taxa	Voucher	Herbarium	Country	Matk	Trnh-Psba	Trnl-F
Lindsaea agatii (Brack.) Lehtonen & Tuomisto	Chen SITW5698	TAIF [481048]	Solomon Islands	**PQ149394**	**PQ149410**	**PQ149412**
Lindsaea blotiana K.U.Kramer	Rakotondrainibe 6350	P	Madagascar	–	GU478510	GU478813
Lindsaeaensifolia Sw.	Fraser-Jenkins FN29	TAIF [357318]	Nepal	–	**PQ149405**	–
Lindsaeaensifolia Sw.	Fraser-Jenkins FN36	TAIF [357830]	India	–	**PQ149404**	–
Lindsaeaensifolia Sw.	Huang 1657	TAIF [307881]	Cambodia	–	**PQ149407**	–
Lindsaeaensifolia Sw.	Kessler 13597	UC [1951608]	Malaysia	–	GU478521	–
Lindsaeaensifolia Sw.	Larsen 41363	AAU	Thailand	–	GU478522	GU478853
Lindsaeaensifolia Sw.	Lu 15719	TAIF [398238]	Thailand	**PQ149396**	**PQ149403**	**PQ149416**
Lindsaeaensifolia Sw.	Lu 16172	TAIF [296864]	Bangladesh	–	**PQ149408**	–
Lindsaeaensifolia Sw.	Chen SITW5235	TAIF [480899]	Solomon Islands	**PQ149399**	**PQ149402**	**PQ149415**
Lindsaeaensifolia Sw.	Chen Wade1339	TAIF [411090]	Vietnam	**PQ149398**	**PQ149401**	**PQ149414**
Lindsaeaensifolia Sw.	Chen Wade1399	TAIF [411162]	Vietnam	**PQ149397**	**PQ149406**	**PQ149417**
Lindsaea fraseri Hook.	Streimann 8951	L	Australia	–	FJ360908	FJ360998
Lindsaea heterophylla Dryand.	Kramer 8285	Z	China	–	KF652043	KF652056
Lindsaea javanensis Blume	Chen Wade4151	TAIF [458804]	Vietnam	**PQ149393**	**PQ149409**	**PQ149411**
Lindsaeakohkongensis I.C.Hwang, M.O.Moon & B.Y.Sun	Chen Wade5034	TAIF [514046]	Vietnam	**PQ149395**	**PQ149400**	**PQ149413**
Lindsaea leptophylla Baker	Raharimalala 2017	MO	Madagascar	–	GU478509	GU478819
Lindsaea madagascariensis Baker	Rakotondrainibe 6349	P	Madagascar	–	GU478512	GU478815
Lindsaea media R.Br.	van der Werff 11655	MO	Australia	–	GU478516	GU478843
Lindsaea meifolia (Kunth) Mett. ex Kuhn	Liesner 7062	MO	Venezuela	–	GU478478	GU478783
Lindsaea orbiculata (Lam.) Mett. ex Kuhn	Averyanov et al. VH4814	AAU	Vietnam	–	FJ360922	FJ361013
Lindsaea oxyphylla Baker	Gautier 2662	P	Madagascar	–	GU478508	GU478816
Lindsaea vieillardii Mett.	McKee 5304	U	New Caledonia	–	GU478496	GU478844
Lindsaea walkerae Hook.	Bostock 638	Z	Australia	–	GU478517	GU478841
Tapeinidium luzonicum (Hook.) K.U.Kramer	Kessler 13610	GOET	Malaysia	–	GU478444	GU478751

**Table A2.** Specimens of *Leptochilus* used for molecular phylogenetic analysis in this study. New sequences generated in this study are indicated in bold.

Taxa	Voucher	Herbarium	Country	Rbcl	Rps4-Trns	Trnl-F
Leptochilusaxillaris (Cav.) Kaulf.	Wu 2462	KUN	Laos	JX103701	JX103743	JX103785
Leptochiluscantoniensis (Baker) Ching	Dong 743	PE	China	EU482945	EU482995	EU483041
Leptochiluscantoniensis (Baker) Ching	Dong 172	PE	China	EU482946	EU482996	EU483042
Leptochiluscantoniensis (Baker) Ching	Kuo 1701	TAIF [509328]	China	MT137055	MH665095	MH665162
Leptochilusdecurrens Blume	Chen Wade1769	TAIF [388951]	Indonesia	MH768462	MH768527	MH768586
Leptochilus digitatus (Baker) Noot.	Chao 1556	TAIF (432480)	Vietnam	MH665034	MH665097	MH665164
Leptochilus dissimilialatus (Bonap.) Liang Zhang & Li Bing Zhang	Zhang et al. 6362	CDBI, MO, VNMN	Vietnam	MH768419	MH768481	MH768547
Leptochilusellipticus (Thunb.) Noot.	Chen Wade3656	TAIF [440115]	Japan	MH768444	MH768508	MH768572
Leptochilus evrardii (Tardieu) Liang Zhang & Li Bing Zhang	Zhang et al. 8633	CDBI, MO	Vietnam	MH768461	MH768526	MH768585
Leptochilus flexilobus (Christ) Liang Zhang & Li Bing Zhang	Chen Wade2518	TAIF [440142]	China	MH665042	MH665106	MH665173
Leptochilus henryi (Baker) X.C.Zhang	Zhang et al. 9289	CDBI	China	MH768459	MH768524	MH768583
Leptochilus heterophyllus (S.K.Wu & P.K.Lôc) comb. ined.	Wu WP-135	KUN	Vietnam	JX103688	JX103730	JX103772
Leptochilus longipes (Ching) X.C.Zhang	Chen Wade4102	TAIF [458794]	Vietnam	MH665049	MH665114	MH665181
Leptochilus macrophyllus (Blume) Noot.	Chen Wade1962	TAIF [388897]	Indonesia	MH768449	MH768513	MH768575
Leptochilus oblongus Li Bing Zhang, Liang Zhang & N.T.Lu	Zhang et al. 6299	CDBI, MO, VNMN	Vietnam	MH768429	MH768491	MH768557
Leptochilus pedunculatus (Hook. & Grev.) Fraser-Jenk.	Chen Wade1334	TAIF [410768]	Vietnam	MH051168	MH113467	MH113500
Leptochilus pentaphyllus (Baker) Liang Zhang & Li Bing Zhang	Zhang 1777	KUN	China	MH768474	MH768539	MH768599
Leptochiluspoilanei (C.Chr. & Tardieu) Liang Zhang & Li Bing Zhang	Chen Wade6804	TAIF	Vietnam	**PQ149390**	**PQ149391**	**PQ149392**
Leptochiluspothifolius (Buch.-Ham. ex D.Don) Fraser-Jenk.	Chen Wade2519	TAIF [440141]	China	MH665056	MH665122	MH665189
Leptochilus pteropus (Blume) Fraser-Jenk.	Chen 1010	H	Taiwan	MH051176	MH113475	MH113508
Leptochilus saxicola (H.G.Zhou & Hua Li) Liang Zhang & Li Bing Zhang	Wei 2017	KUN	China	MH768471	MH768536	MH768595
Leptochilus wrightii (Hook.) X.C.Zhang	Chen 1087	H	Taiwan	MH051170	MH113469	MH113502
